# It’s awe-fully unfamiliar: The effect of familiarity on awe within a virtual reality setting

**DOI:** 10.3389/fpsyg.2023.1096283

**Published:** 2023-02-22

**Authors:** Cassidy Ochadleus, Charissa Kirby, Christie Napa Scollon

**Affiliations:** Department of Psychology, Western Washington University, Bellingham, WA, United States

**Keywords:** awe, accommodation, positive affect, positive psychology, nature, virtual reality

## Abstract

Awe-inducing scenes are purported to involve vastness and a need for accommodation. Familiarity with a stimulus should reduce the need for accommodation, thereby reducing the intensity of awe experienced. The present study tested the effect of familiarity to a natural awe-inducing scene on the experience of awe. Forty undergraduate participants (*N* = 40) participated in a virtual reality experiment. Using a within-subjects design, participants viewed (in counterbalanced order) an awe-inducing nature scene that was either familiar or unfamiliar. The dependent measure was self-reported awe. Results confirmed that participants experienced diminished awe while viewing a familiar awe-inducing scene compared to viewing an unfamiliar awe-inducing scene.

## 1. Introduction

*I never knew--I never imagined what mountains were before. The immensity of these aerial summits excited, when they suddenly burst upon the sight, a sentiment of ecstatic wonder, not unallied to madness. And remember this was all one scene, it all pressed home to our regard and our imagination. Though it embraced a vast extent of space, the snowy pyramids which shot into the bright blue sky seemed to overhang our path; the ravine, clothed with gigantic pines, and black with its depth below, so deep that the very roaring of the untamable Arve, which rolled through it, could not be heard above--all was as much our own, as if we had been the creators of such impressions in the minds of others as now occupied our own* (Mary Shelley, as cited in Kelly, 2022).

Writers have long been enamored with the beauty of nature, and many have expressed awe at the sweeping scenes from atop majestic mountains as Mary Shelley did when viewing Mount Blanc in the Alps for the first time (Kelly, 2022). Awe is a self-transcendent emotion (Nelson-Coffey et al., 2019) often described as a feeling of wonder and amazement at something that seems larger than life. Awe is more than being amazed, and it is more than a feeling of inspiration or astonishment (although it can certainly elicit all these). Instead, awe involves a profound shift in cognition, a rewriting (or perhaps rewiring) of one’s cognitive framework, that occurs in the presence of something powerful and vast, something that illuminates the smallness of oneself.

Jean Piaget initially theorized that when we encounter new information and experiences that do not fit with our current structures for understanding, cognitive schemas, this creates an imbalance which is resolved by a process called equilibration. When new information fits into existing mental structures, balance is restored through assimilation. When our understanding is changed with the creation of new schemas and the adaptation of existing ones, balance is restored through accommodation (Cohen and Kim, 1999). Awe-inducing experiences demand accommodation.

With specific regard to awe, Keltner and Haidt (2003) define accommodation as the inability to integrate an experience with current mental structures. They describe it as an essential feature of awe as the emotion stems from our mental structures’ failure to make sense of an experience. As such, awe creates a need for accommodation, which may remain unresolved.

The natural world is one place where we can easily find awe-inducing experiences because they are both perceptually vast and create a need for accommodation (Keltner and Haidt, 2003; Shiota et al., 2007). While awe is not only experienced in the presence of nature—for example, people in positions of power and grand architecture can also create feelings of awe—nature is one of the most common elicitors of awe both in everyday life and in experimentally induced settings (Shiota et al., 2007; Piff et al., 2015; Ballew and Omoto, 2018).

Given that awe-inducing experiences demand accommodation, what is the effect of repeated experience with an awe-inducing stimulus? With environments that are moderately novel, past experiences already provides a basis for understanding which individuals then use for assimilation (Cohen and Kim, 1999). In other words, greater exposure to a particular awe-inducing scene would lead to more developed cognitive schemas, and a lower need for accommodation. Understanding the effect of familiarity on awe is important because while awe has been demonstrated to have significant prosocial/interpersonal benefits (Shiota et al., 2007; Rudd et al., 2012; Piff et al., 2015; Nelson-Coffey et al., 2019; Perlin and Li, 2020; Stancato and Keltner, 2021), seeking out awe inspiring natural scenes to induce awe would have limited efficacy if familiarity diminishes awe. With globalization and the ability to share high resolution digital images from anywhere in the world at the touch of a button, people may be becoming more familiar with nature’s wonders from afar. With increasing familiarity, these scenes may produce less intense experiences of awe.

The present study is the first to examine the effect of familiarity to the stimulus on the experience of awe. We propose that greater familiarity will result in lower intensity of awe. The study also makes a contribution to the awe literature by using virtual reality to experimentally manipulate awe. Virtual reality has been verified as a valid means of inducing awe in a controlled laboratory setting (Chirico et al., 2017; Quesnel and Riecke, 2018; Nelson-Coffey et al., 2019), but remains relatively rare in emotion-induction experiments. Past research has suggested that virtual reality is better for experimentally inducing awe than two-dimensional images (Chirico et al., 2017). While other studies have manipulated awe by bringing participants to awe inspiring nature scenes (Piff et al., 2015; Ballew and Omoto, 2018), in doing so, researchers often sacrifice controllability in exchange for ecological validity. The use of two-dimensional Google Earth VR images over other three-dimensional VR images in this study ensured standardization across conditions. The images chosen were of specific locations without watermarks, people, animals, or other things that may have distracted the participant from the scenery. While past studies have used three-dimensional images on Google Earth VR (Quesnel and Riecke, 2018), this introduces an interactive element to the VR experience (allowing participants to move around). For this study we did not want participants to change the setting they were in.

### 1.1. Overview

The current study used virtual reality images to experimentally manipulate familiarity and measure the effect it had on participants’ experience of awe. We hypothesized that increased familiarity to the stimulus would decrease the intensity of awe. This is based on the idea that repeated exposure to awe inducing scenes would decrease the need for accommodation.

## 2. Methods

### 2.1. Participants

The study was preregistered on OSF.^1^ A sample of 50 undergraduate psychology students (28 female, 13 male, 8 non-binary, 1 other). Age ranged from 18 to 57 (*M* = 21.74, *SD* = 6.82). Participants were recruited from Western Washington University to participate in this study. Subjects were recruited through the undergraduate Psychology subject pool and were told that the study was on “emotional responses to virtual reality.” Participants received course credit as an incentive for participation.

Using G*Power 3.1 (Faul et al., 2009), we conducted *a priori* power analysis to determine the minimum number of subjects needed. Based on an estimated effect size of 0.40 with alpha set to 0.05 and power set to 0.80, the minimum number of subjects required for a within-subjects design was 52 in total (26 per condition). We aimed to collect data on 60 participants or until our *a priori* cut-off date.

The final sample size was *N* = 40 after removing a total of 10 subjects from the dataset. One participant was removed because the virtual reality headset failed while they were viewing one of the awe scenes. The other nine subjects were removed because they were either too familiar with the unfamiliar condition (eight subjects) or too unfamiliar with the familiar condition (one subject), which was determined by a threshold that was set before data collection and recorded in our preregistration (see Measures for details).

### 2.2. Materials

The study used an HTC Vive virtual reality headset located in a virtual reality lab on the Western Washington University campus. The participants each viewed three virtual reality images in total, all from the Google Earth VR application. All the images were two-dimensional, 360-degree photos (see for original materials). Participants sat on an office chair (one capable of rotating) in the center of the small room where they were able to view the images with the headset. One of the images used was a picture of a flower garden from Fort Vancouver Garden in Vancouver, WA. This was a neutral image, not a true condition, and was used only to allow participants to acclimate to the virtual reality setting and help the researcher adjust the headset before the participant viewed the first condition. As such, this location was chosen because, while it is pretty, it does not possess the qualities which make a scene inherently awe inducing.

The image used for the awe-familiar condition was a picture of an old growth forest taken on a trail near Baker Lake in Bellingham, WA. The image used for the awe-unfamiliar condition was a picture of the Grand Canyon in Arizona. Both tall, old growth trees (Piff et al., 2015; Chirico et al., 2017) and the Grand Canyon (Gill et al., 2022) are perceptually vast natural scenes that fit with established features of awe (Keltner and Haidt, 2003; Shiota et al., 2007). The images were chosen specifically so participants familiarity with the scenes would differ based on the condition. All our participants were recruited from Western Washington University in Bellingham, WA. As such most of them are from the Pacific Northwest, so we determined that they should be more familiar with tall trees from that area than the Grand Canyon, something we verified with a manipulation check. Additionally, the trees in the image resembled the ones in the arboretum on the Western Washington University campus.

### 2.3. Measures

Participants responded to an awe scale previously developed and used by Collado and Manrique (2020) to measure the intensity of awe experienced after viewing each condition (awe-familiar α = 0.63; awe-unfamiliar α = 0.44.) The scale consisted of 10 items meant to measure different aspects of awe (e.g., “This image makes me feel that I’m part of something much larger than myself.” and “When I look at this image, it feels as if time stopped.”). Participants responded to the items using a 1 (strong disagree) to 7 (strong agree) scale. Thus, higher scores on the scale indicated a greater intensity of awe experienced.

The study also included a manipulation check for familiarity to the stimulus. Subjects responded to two items (“How familiar are you with environments like the image you viewed?” and “I have spent a lot of time in environments that look like this.”) intended to verify that they were more familiar with the familiar condition and less familiar with the unfamiliar condition (awe-familiar α = 0.88; awe-unfamiliar α = 0.69). The first item used a 7-point semantic differential scale (1 = Unfamiliar and 7 = Familiar) and the second item used a 7-point Likert-type scale to measure familiarity to the stimuli. Higher scores on the items indicated more familiarity with the stimulus. Participants whose combined responses on these two items was 12 or higher for the awe-unfamiliar condition and 3 or lower for the awe-familiar condition were removed from analysis. This threshold was set prior to data collection to ensure each condition would reflect the intended manipulation of familiarity with the stimulus (see^2^).

### 2.4. Procedure

The study used a within-groups design which allowed us to directly compare awe experienced by each individual across each condition. The awe conditions were counterbalanced to control for any possible order effects. Participants were randomly assigned to view either the awe-familiar condition first and the awe-unfamiliar condition second or the awe-unfamiliar condition first and the awe-familiar condition second. Each participant was randomly assigned to a condition order using a six-sided die with three blue sides and three red sides, where the blue side indicated participants would view the awe-familiar condition first and the red side indicated they would view the awe-unfamiliar condition first.

Participants came to the virtual reality lab, and after informed consent was obtained, they were asked to sit down on a chair in the center of the room. First, every participant viewed a neutral image of a garden with the VR headset for 2 mins. The purpose of this was to give the researcher time to make sure the VR headset was on correctly and to give the participant time to adjust to the format. Additionally, participants were instructed beforehand to inform the researcher immediately if they felt any discomfort or nausea from the headset. Viewing this image first gave them time to see if they would experience discomfort before moving on to the conditions. None of the participants experienced any discomfort from the virtual reality environment. Before viewing each awe image, participants were instructed to “pay attention to the details of [their] surroundings and allow [themselves] to become absorbed in the experience.” These instructions were intended to help control for the possibility that people may pay more attention and be generally more absorbed in unfamiliar scenes. Absorption has been demonstrated to be a moderator of awe (Ballew and Omoto, 2018) so these instructions were given before each condition to maximize absorption. Next, participants viewed either the awe-familiar or the awe-unfamiliar image for 5 mins with the VR headset before filling out a 10-item survey measuring the intensity of awe experienced and a two-item manipulation check measuring familiarity to the stimulus. Then, participants viewed the image for the condition they had not seen yet for 5 mins with the VR headset before filling out the survey again as well as some demographics questions at the end. Finally, participants were thanked and debriefed.

## 3. Results

The results of our manipulation check showed that participants were indeed more familiar with the awe-familiar image (*M* = 6.4, *SD* = 0.83) compared to the awe-unfamiliar image [*M* = 2.8, *SD* = 1.26; *t* (39) = 17.19, *p* < 0.001]. This confirmed that our experimental manipulation of familiarity was successful.

We used a two-tailed paired samples *t*-test to compare participants’ mean awe score in the awe-familiar condition to their mean awe score in the awe-unfamiliar condition. Results indicated that participants in the awe-unfamiliar condition (*M* = 4.6, *SD* = 0.63) experienced significantly greater levels of awe compared to the awe-familiar condition [*M* = 4.0, *SD* = 0.74; *t* (39) = 5.69, *p* < 0.001]. These results support our hypothesis that increased familiarity to an awe inducing stimulus would decrease the intensity of awe experienced.

We counterbalanced for order to control for the possibility that there could be an effect of seeing one image first. A 2 (order) × 2 (familiarity) repeated measures ANOVA with familiarity as the within-subjects factor confirmed there were no effects of order *F* (1, 38) = 2.208, *p* = 0.146 and no interactions among order and familiarity on awe *F* (1, 38) = 0.006, *p* = 0.936.

## 4. Discussion

This study used experimental manipulation to demonstrate that familiarity does diminish the intensity of awe experienced when looking at awe inspiring natural scenes. To the best of our knowledge, this is the first study to establish the effect of familiarity on the experience of awe. These results were unlikely due to differences in immersion to the scenes given that participants were explicitly instructed to allow themselves to become absorbed in the experience across both conditions. The use of virtual reality, careful selection of stimuli, and detailed participant instructions controlled for other factors that can influence an awe experience allowing us to zero in on the effects of familiarity to specific scenes.

Nevertheless, it remains possible that there are other aspects of familiarity that could contribute to different awe responses which were not addresses by the current study and may warrant further investigation. For example, some people might approach a familiar awe stimulus with the intent to experience it in a new light, perhaps even seeking to find unfamiliar elements within it. We recognize that awe is a complex phenomenon with multiple factors shaping the experience.

Although we did not measure accommodation directly, the present findings have interesting implications for the notion that awe creates a need for accommodation. This study suggests that perhaps repeated exposure to an awe inducing stimulus lessens the need for accommodation over time by solidifying a person’s existing cognitive schema for the stimulus.

Past research has indicated that the left middle temporal gyrus (MTG) plays an important role in matching one’s existing schemas to an event they are experiencing (Davey et al., 2016; Takano and Nomura, 2022). Recently, Takano and Nomura (2022) identified the neural mechanisms of awe. They found that awe experiences (both positive and threat awe) led to decreased activation in the left MTG. These results, combined with past research, suggest that decreased activation of the left MTG is a neural mechanism for accommodation. Future research could extend the idea presented in the current study by examining participants neural activity after repeated viewing of the same awe inducing stimulus, paying particular attention to activity in the left MTG.

Awe is a complex and important emotion. As such it is important to understand how it operates within us and what factors affect it. Past research has suggested that awe increases prosocial behavior in a variety of ways. This is due in part to awe’s ability to diminish one’s sense of self and create feelings of being part of a larger whole, while generally shifting focus from the self to others (Shiota et al., 2007; Piff et al., 2015; Nelson-Coffey et al., 2019; Perlin and Li, 2020). Additionally, there is research to suggest that awe slows the perception of time passing creating the illusion of having more time (Rudd et al., 2012). Future studies on awe and familiarity would benefit from measuring these potential mediators.

The effects of awe have been linked to increased helping behavior, generosity, support for ethical decisions, and prosocial values (Piff et al., 2015) as well as increased willingness to volunteer time (Rudd et al., 2012). A separate, but related, effect of awe which has been studied more recently by Stancato and Keltner (2021) suggests that it can also lessen certainty in ideological convictions and increase a sense of cohesion. Essentially, awe is incredibly beneficial for maintaining positive interpersonal relationships and making it easier to live together as members of a shared community.

### 4.1. Limitations and caveats

Although this was the first study of its kind to demonstrate the effect of familiarity on awe, the study was not without its limitations. First, we were unable to conduct a pilot study with the images used in the study. Therefore, we cannot be certain that the two images were equivalent in terms of vastness, beauty, and other characteristics. Second, although we suspect that accommodation mediates the process by which familiar scenes affect awe, we did not assess accommodation. Future work should confirm this process through mediational analyses.

## Data availability statement

The raw data supporting the conclusions of this article will be made available by the authors, without undue reservation.

## Ethics statement

The studies involving human participants were reviewed and approved by Western Washington University Internal Review Board. The patients/participants provided their written informed consent to participate in this study.

## Author contributions

CO designed the study, conducted analyses, and wrote the first draft of the manuscript. CK assisted with research design and data collection. CS assisted with research design, data analysis, and commented on the manuscript. All authors contributed to the article and approved the submitted version.

## Conflict of interest

The authors declare that the research was conducted in the absence of any commercial or financial relationships that could be construed as a potential conflict of interest.

## Publisher’s note

All claims expressed in this article are solely those of the authors and do not necessarily represent those of their affiliated organizations, or those of the publisher, the editors and the reviewers. Any product that may be evaluated in this article, or claim that may be made by its manufacturer, is not guaranteed or endorsed by the publisher.

## Footnotes

^1^ https://osf.io/hsbgw

^2^ https://osf.io/hsbgw
